# Prescription and use of psychoactive medications among cancer patients and associated factors in lower and upper middle-income countries: systematic review

**DOI:** 10.1007/s00520-025-10272-8

**Published:** 2026-01-06

**Authors:** Tassadit Merabtine, Dieu donné Gnonlonfoun, Elodie Marcellaud, Sarah Altayyar, Zeinab Tarhini, Niki Christou, Jeremy Jost

**Affiliations:** 1 Inserm U1094, IRD UMR270, Univ. Limoges, CHU Limoges, EpiMaCT - Epidemiology of Chronic Diseases in Tropical Zone, Institute of Epidemiology and Global Health – Michel Dumas, Omega Health, Limoges, France; 2 Laboratory of Epidemiology of Chronic and Neurological Diseases (LEMACEN), FSS/EDSS, University of Abomey-Calavi, Cotonou, Benin; 3 Department of neurology, CNHU-HKM, Cotonou, Benin; 4 Laboratory INSERM U1308, CAPTuR, Control of Cell Activation in Tumor Progression and Therapeutic Resistance, Medical School, 2 Rue du Docteur Marcland, 87025 LIMOGES Cedex, France; 5 Digestive Surgery Department, University Hospital of Limoges, Avenue Martin Luther King, 87000 Limoges, France; 6 Clinical Pharmacy Unit, Pharmacy Department, University Hospital of Limoges, Avenue Martin Luther King, 87000 Limoges, France

**Keywords:** Psychoactive medication, Prescription patterns, Cancer patients, Low- and middle-income countries (LMICs)

## Abstract

**Purpose:**

To describe the prescription and use of psychoactive medications among cancer patients in low- and middle-income countries (LMICs) and explore the factors influencing their prescription and use.

**Methods:**

This systematic review was conducted following the PRISMA guidelines. Six electronic databases were searched. We included observational studies that investigated the prescription and use of psychoactive medications among adult cancer patients in low- and middle-income countries (LMICs) according to the 2024 World Bank’s classification. Eligible studies specifically focused on the use of these medications for the management of psychological disorders in this population.

**Results:**

Ten studies were included in the systematic review. Psychoactive medications prescribed were antidepressants, benzodiazepines, non-benzodiazepine/Z-drugs, and antipsychotics (both typical/first-generation and atypical/second-generation). Several factors, such as comorbidities, polypharmacy, geographical disparities, and health insurance coverage, influenced the prescription and use of these medications. Potentially inappropriate use of antidepressants and benzodiazepines was observed, particularly among elderly patients with multimorbidity. Underdiagnosis and undertreatment of mental health disorders were also reported, often leading to inadequate management of psychological distress in cancer patients.

**Conclusion:**

In conclusion, this review highlights the complexity of prescribing and using psychoactive medications among cancer patients. Their use is influenced by multiple factors, including comorbidities, polypharmacy in older adults, and socioeconomic disparities that affect access to healthcare.

**Supplementary Information:**

The online version contains supplementary material available at 10.1007/s00520-025-10272-8.

## Introduction

Cancer is a leading cause of both morbidity and mortality worldwide, imposing a growing health burden, especially for low- and middle-income countries (LMICs). These regions face a dual health challenge. As infectious diseases still contribute heavily to morbidity and mortality, the incidence of non-communicable diseases, including cancer, is highly increasing. According to the World Health Organization (WHO), over 70% of cancer-related deaths occur in LMICs, where access to specialized care and treatment remains limited. Projections suggest that by 2030, nearly three-quarters of all cancer deaths will occur in these regions [1, 2].

Beyond the physical suffering caused by cancer, patients frequently experience psychological distress, including anxiety, depression, and other mental health disorders, which can further compromise their overall condition [3, 4]. Even without a formal mental health diagnosis, nearly all cancer patients experience some degree of psychological distress at different stages of the disease, which can negatively impact their quality of life (QOL) and is associated with increased mortality [5]. A study conducted by Walker et al. found that the prevalence of depression and anxiety among cancer patients tends to be higher in low- and lower-middle-income countries compared to high-income countries [6]. Similarly, a study conducted in Africa revealed high rates of mental health disorders among cancer patients, with pooled prevalence estimates of 53.21% (95% CI: 47.47–58.94) for depression and 53.32% (95% CI: 46.85–59.80) for anxiety, highlighting a significant mental health burden in these populations. In this meta-analysis, self-administered questionnaires such as HADS, PHQ, and the Beck Depression Inventory were used to assess depression and anxiety, which may have contributed to higher prevalence estimates [7].

Moreover, a recent meta-analysis showed that depression after a cancer diagnosis is associated with a significantly increased risk of cancer-specific mortality across multiple cancer types [8].

Given the significant impact of mental health on cancer patients, it is essential to address their psychological needs, especially in low- and middle-income countries (LMICs), where recognition resources are limited, and mental health care is often overlooked. Despite the growing need for psychosocial support, the mental health burden among cancer patients in these regions has yet to be systematically assessed [6]. Furthermore, access to mental health services remains limited. Several barriers, such as the strong stigma surrounding mental health issues, a shortage of trained mental health professionals, the absence of mental health services within oncology care systems, and limited healthcare infrastructure, make it difficult for patients to access the psychological support they need [9]. The lack of integration and collaboration between mental health and cancer care services further exacerbates these challenges. As a result, many cancer patients in LMICs experience untreated psychological disorders, which can severely affect their overall well-being [10, 11].

Appropriate care for psychological disorders in cancer patients includes psychotherapy as the first-line approach, with pharmacological treatment considered in cases of severe or psychotic symptoms, when psychotherapy is not effective, or when access to specialized psycho-oncology services is limited [12]. However, the prescription and use of psychoactive medications among cancer patients face significant challenges, particularly in LMICs. One important issue is the frequent late diagnosis of cancer, which complicates the overall management of both cancer and mental health [13]. Additionally, there is often a lack of monitoring regarding the medications that cancer patients are taking, which may lead to potential drug interactions, adverse effects, and potentially inappropriate use [14].

Despite the important role of psychoactive medications in managing mental health issues, there is a lack of comprehensive data on the prescribing and use of these medications among cancer patients in LMICs. This knowledge gap limits our ability to better understand disparities in access to psychological care and associated socio-economic factors. Understanding these patterns is important for identifying existing challenges and developing adapted strategies to improve mental care among cancer patients in resource-limited settings.

This study aimed to describe the prescription and use of psychoactive medications among cancer patients in LMICs and explore the factors influencing their prescription and use.

## Methods

This systematic review was conducted following the PRISMA (Preferred Reporting Items for Systematic Reviews and Meta-Analyses) guidelines [15]. The study protocol was registered in the International Prospective Register of Systematic Reviews (PROSPERO) under the identification number CRD42024560300.

### Search strategy

Searches were conducted across six databases including PubMed, Scopus, Google Scholar, SciELO, AJOL, and APA PsychArticles. The search terms focused on keywords related to prescription and medication use, and psychoactive medications in the context of cancer. The focus was also on studies from low- and middle-income countries (LMICs) according to the World Bank’s 2024 classification. In addition to the database searches, a manual search of relevant references was performed to ensure large coverage of the topic.

### Study selection

The selection process adhered to predefined inclusion and non-inclusion criteria. Eligible studies were those conducted in low- and middle-income countries (LMICs), focusing on the use of psychoactive medications for the treatment of clearly defined psychological disorders in adult cancer patients. The medications considered belonged to the following Anatomical Therapeutic Chemical (ATC) Classifications: N05 “Psycholeptics” (N05A: Antipsychotics, N05B: Anxiolytics, N05C: Hypnotics and sedatives) and N06A (Antidepressants) according to the WHO Collaborating Centre for Drug Statistics Methodology.

Longitudinal and cross-sectional studies were included. Studies were excluded if they were systematic reviews, meta-analyses, abstracts, editorials, or case reports. Further exclusions applied to studies involving non-cancer patients, cancer patients not residing in LMICs, or those not receiving psychoactive medications.

### Study quality assessment

The quality of the selected studies was independently assessed by two authors using the Newcastle–Ottawa Scale (NOS) for cohort studies, and its adapted version for cross-sectional studies [16, 17]. These tools were specifically designed for each study type, focusing on three key domains: selection of study groups, comparability between groups, and assessment of outcomes. Cohort studies could receive a maximum of 9 points across these domains, while cross-sectional studies could receive a total of 10 points.

Any disagreements between the two assessors during the quality assessment process were resolved through discussion and consensus. If a consensus could not be reached, a third reviewer was consulted to make the final decision.

### Data extraction

For each study, the following data were extracted:

- Study characteristics: including the publication year, authors’ names, country of the study, study design, sample size, and study objectives.
- Socio-demographic variables: including population characteristics such as age, gender, the study settings, which were classified as either population-based or hospital-based studies, income level, and health insurance (when reported).
- Clinical characteristics: cancer types and their prevalence (when reported), identified mental health disorders within the study population, classes and molecules of psychoactive medications, their prescription and use patterns, and medical data sources to assess the reliability of the information.

## Results

### Study selection

**Fig. 1:**
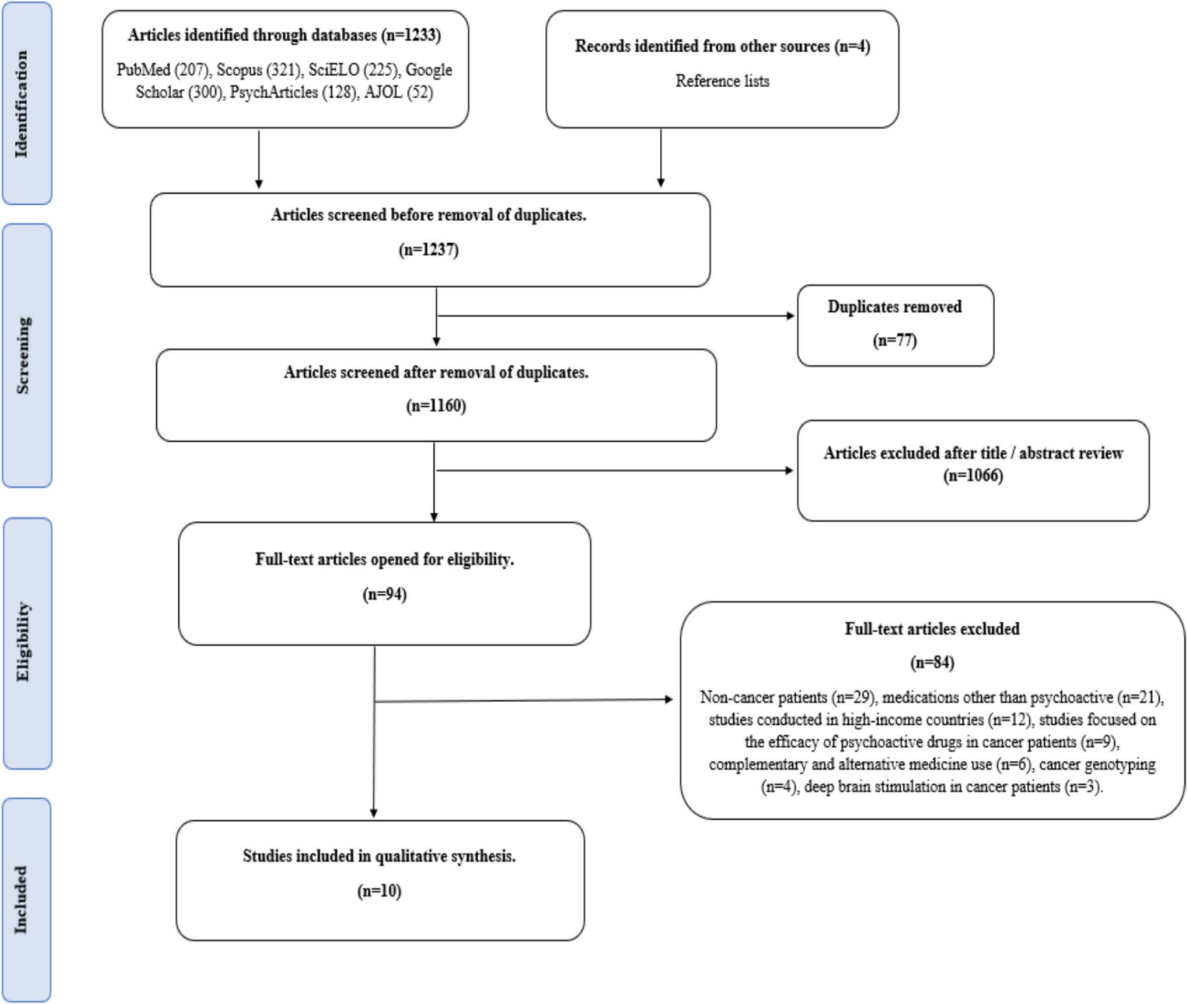
Flowchart summarizing study identification and selection

A total of 1237 articles were identified through the search strategy. After removing duplicates, 1160 articles were retained for further evaluation. Of these, 1066 were deemed irrelevant based on a review of their titles and abstracts. The remaining 94 articles were retrieved and assessed in full text, leading to the exclusion of 84 articles. In total, 10 articles met the inclusion criteria and were included in the review [13, 18–26]. The flowchart for the selection and inclusion of studies is presented in (Fig.1).

### Quality assessment

The quality of the included studies, consisting of two cohort studies and eight cross-sectional studies, was evaluated using the Newcastle–Ottawa Scale (NOS).

The cohort studies demonstrated a good methodological quality, receiving scores of 7 and 8 out of 9. Cross-sectional studies were rated between 6 and 8 out of 10. Most studies showed good performance in the selection and outcome domains reflecting appropriate sampling methods and reliable outcome measurements. However, comparability between study groups was the weakest area, as many studies lacked sufficient information on how potential confounding variables were controlled. Overall, some studies achieved good scores [7, 8], while others were fairly scored 6 out of 10 (Supplementary Material 1).

### Characteristics of included studies

The included studies comprised two cohort and eight cross-sectional studies conducted in middle-income countries, including China, India, Brazil, and Malaysia. Notably, no studies conducted in low-income countries were identified in the review. These studies mainly focused on patients with breast, lung, digestive, and prostate cancers, with the majority being hospital-based. The most commonly prescribed and used psychoactive medications were antidepressants, benzodiazepines, antipsychotics, and hypnotics, primarily used to treat depression, anxiety, and sleep disorders (Table 1).

**Table 1:** General characteristics of included studies

Variables	Details
Total number of studies (n)	10
Study design (n)	Cohort (2), Cross-sectional (8)
Countries	China, India, Brazil, Malaysia
Population-based study (n)	2
Hospital-based study (n)	8
Sample size (n)	48 111 (max)/56 (min)
Gender distribution (%)	Female: ~ 54%, Male: ~ 46%
Most common cancer types	Breast cancer, lung cancer, digestive cancers, prostate cancer
Mental disorders observed	Depression, anxiety, sleep disorders
Prescribed and used psychoactive drugs	Benzodiazepines, antidepressants antipsychotics, hypnotics

*min* minimum, *max* maximum

Additional details regarding study objectives, medical data sources, cancer types, health insurance, and income levels were provided in (Supplementary Material 2).

**Fig. 2:**
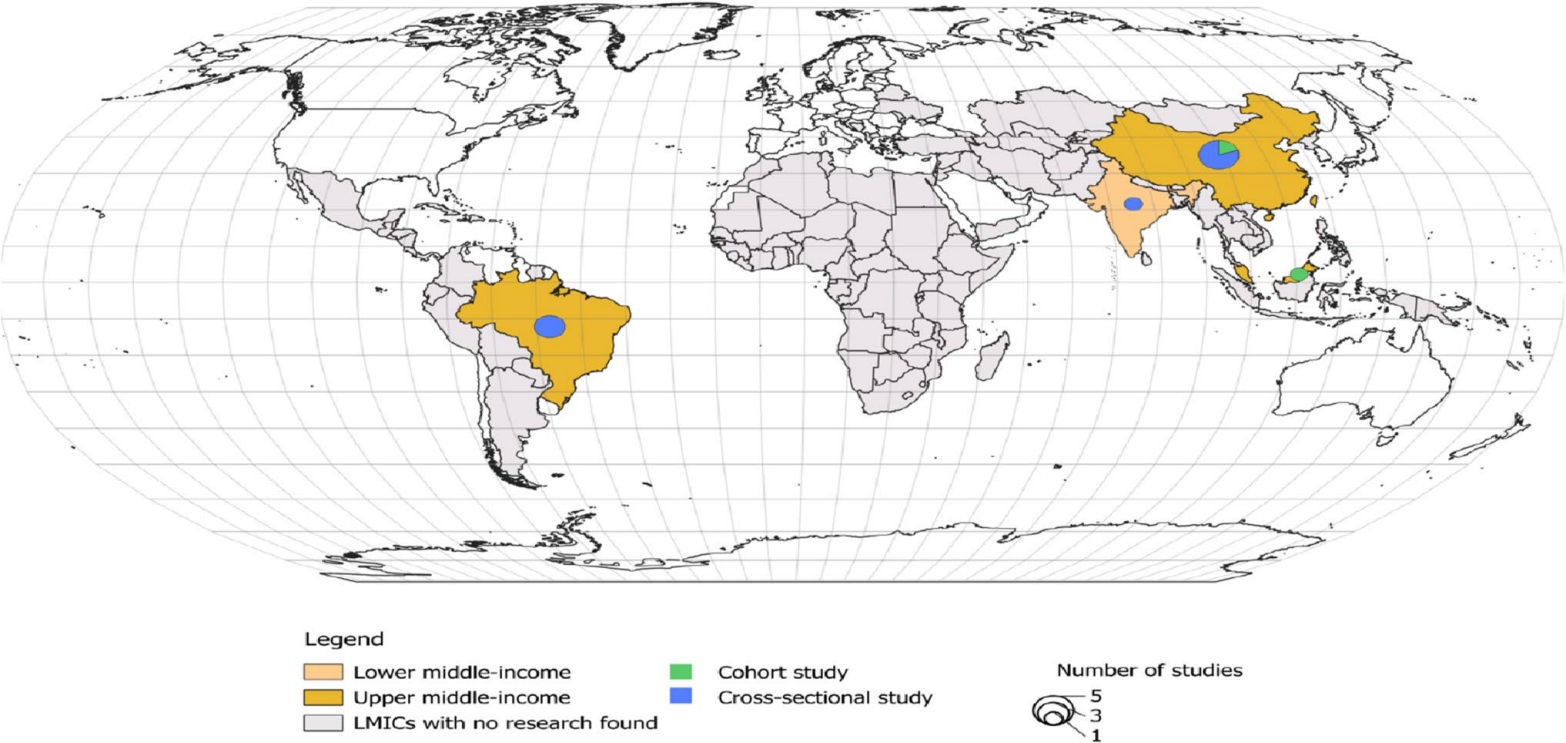
Geographic distribution, income classification and study design of included studies

The geographic distribution, income classification, and study design of the included studies were shown in (Fig. 2).

### Psychoactive medications prescribed and used among cancer patients

The studies included in this synthesis revealed that a variety of psychoactive drugs were commonly prescribed to cancer patients for managing mental health conditions, including anxiety, depression, sleep disturbances, and other psychotic disorders. The prescribed medications were antidepressants (Serotonin reuptake inhibitors (SSRIs), Serotonin and Norepinephrine Reuptake Inhibitors (SNRIs), Tricyclic antidepressants (TCAs), and atypical antidepressants), benzodiazepines, non-benzodiazepine/Z-drugs, and antipsychotics (both typical/first-generation and atypical/second-generation). Benzodiazepines like lorazepam, alprazolam, and diazepam were prescribed for anxiety and sleep disturbances. Other related drugs, such as zolpidem and zopiclone, were also used for insomnia. In addition to benzodiazepines, antidepressants, particularly selective serotonin reuptake inhibitors (SSRIs) such as sertraline and fluoxetine, were prescribed and used, alongside amitriptyline, mirtazapine, and other serotonin-norepinephrine reuptake inhibitors (SNRIs) like venlafaxine and duloxetine for depression. Antipsychotics, such as haloperidol (first-generation), and second-generation antipsychotics including quetiapine, olanzapine, risperidone, and aripiprazole, were used for managing psychotic symptoms and acute agitation. Across studies, psychotropic medication use showed marked variability. Some reported frequent use, especially among patients with comorbidities [18], or highlighted potentially inappropriate prescribing (potentially inappropriate medication (PIM) is defined as those in which the risk of adverse events from the drug outweighs the clinical benefits, and in particular, where there is a safer and more effective alternative) [27] of benzodiazepines and Z-drugs [19, 23]. Others noted low or underuse of antidepressants [22, 26], while in certain cases prescriptions were symptom-driven and not based on psychiatric diagnoses [13]. In addition, some studies reported that antidepressants were among the most frequent therapeutic classes of fall risk-increasing drugs (FRIDs) [20] or that about half of patients with depression received appropriate treatment [24]. More details about the studies’ selected populations, the psychoactive medications used and their respective classes, the proportion of patients with psychiatric diagnoses or psychiatric symptoms, and the methods used for their assessment are presented in Table 2.

**Table 2:** Summary of psychoactive medications prescribed and used among cancer patients with psychiatric disorders

Study/country	Population	% of patients with psychiatric diagnosis/psychiatric symptoms	Psychoactive medication classes (%)	Medications	% of patients with psychoactive medication	Diagnostic or assessment method for psychiatric disorders
Lam et al., 2024 China	*n* = 1868 (Cancer patients who were newly prescribed psychotropic medications after cancer diagnosis)	Only 18.6% (*n* = 348/1868) had a documented diagnosis of psychiatric disorders: Depressive disorders (*n* = 142/348, 40.8%) and Adjustment disorders (*n* = 77/348, 22.1%)	*Z-drugs (50.3%) *Serotonin reuptake inhibitors (SSRI), Tricyclic antidepressants (TCAs), and atypical antidepressants (32.8%) *Conventional antipsychotics and Atypical antipsychotics (31.0%) *Benzodiazepines (30.3%)	*Zopiclone, Zolpidem. *Sertraline, Fluoxetine, Amitriptyline, Trazodone *Haloperidol, Quetiapine *Diazepam, Lorazepam	The use of psychotropic medication is common (20%) among patients with cancer Psychotropic prescriptions were more likely prescribed in patients with multiple comorbidities	NI
Mohamed et al., 2024 India	*n* = 125 (Cancer patients registered for palliative care at home)	Anxiety (50%), depressive symptoms (22%) and psychosis (18%)	*Benzodiazepines (56.8) *Tricyclic antidepressant, atypical antidepressant and Serotonin reuptake inhibitors (SSRI) (31.8%) *Atypical antipsychotics (43.1%) *Conventional antipsychotics. (13.6%)	*Alprazolam, Lorazepam, Clonazepam *Amitriptyline, Mirtazapine and SSRIs *Quetiapine, Olanzapine, Risperidone *Haloperidol	Psychotropic medication was used in 35.2% of patients, mostly initiated by palliative care (75%), with psychiatrists involved in only (25%). Prescriptions were largely symptom-driven, not based on psychiatric diagnosis	Most prescriptions (63%) were symptom-driven. Only 36% of prescriptions targeted specific diagnoses
Tian et al., 2022 China	*n* = 6160 (Outpatients (age ≥ 65 years) with cancer and multimorbidity)	NI	*Benzodiazepines *Z-drugs	*Estazolam, Alprazolam, Clonazepam, Diazepam. *Eszopiclone, Zolpidem	One of the most common potentially inappropriate medication used were benzodiazepines and Z-drug hypnotics, accounting for 27.7% of all identified PIMs (*n* = 2427)	NI
Machado et al., 2022 Brazil	*n* = 153 (Patients with multiple myeloma and treated in the oncology and hematology departments of a capital city in south-east Brazil)	Depression (4.6%)	*Serotonin reuptake inhibitors (SSRIs), Serotonin and Norepinephrine Reuptake Inhibitors (SNRIs), Tricyclic antidepressants (TCA), and Atypical antidepressants (20.5%) *Benzodiazepines (5.1%) *Benzodiazepines (5.1%) *Z-drugs (5.1%) * Conventional and Atypical antipsychotics (4.0%)	*Fluoxetine, Escitalopram, Citalopram, Sertraline, Duloxetine, Venlafaxine, Amitriptyline, Imipramine, Nortriptyline, Mirtazapine, Trazodone, Vortioxetine *Clonazepam *Alprazolam, Lorazepam, Bromazepam *Zolpidem * Haloperidol, Chlorpromazine, and Quetiapine, Aripiprazole	Antidepressants were among the most frequent therapeutic classes of fall risk-increasing drugs (FRIDs), accounting for 20.5% of cases	NI
Pu et al., 2022 China	*n* = 152 (Patients with advanced cancer who received antidepressant intervention with Sertraline)	Depression (41.5%) Anxiety (26.3%) Comorbid anxiety and depression (20.4%)	*Serotonin reuptake inhibitors (SSRI) *Serotonin reuptake inhibitors (SSRI) and benzodiazepine	*Sertraline *Sertraline and Diazepam	142 patients had completed sertraline intervention for ≥ 1 week Diazepam was added for 45 patients (31.7%)	Hospital Anxiety and Depression Scale score (HADS) patients were categorized into 4 risk groups: high (HADS score, ≥ 15), medium (10–14 points), low (8–10 points), and no (≤ 7 points) risk
Bai et al., 2020 China	*n* = 48,111 (Cancer patients identified in the insurance database, who were prescribed at least one psychotropic medication)	Mania, anxiety, insomnia and depression	*Conventional antipsychotics and Atypical antipsychotics (1.4%) *Benzodiazepines and non-benzodiazepine anxiolytic (6.1%) *Benzodiazepines and Z-drugs (14.2%) * Tricyclic antidepressants (TCA), Serotonin reuptake inhibitors (SSRI) (0.9%)	*Haloperidol, Droperidol and Chlorpromazine, Olanzapine, Risperidone *Diazepam, Alprazolam, Lorazepam, Oxazepam and Buspirone *Midazolam, Estazolam and Zolpidem, Zopiclone *Amitriptyline, Doxepin, Melitracen and Paroxetine, Escitalopram	The prevalence of psychotropic medication use in Chinese adult cancer patients was 18.5%	NI
Reis et al., 2017 Brazil	*n* = 160 (Patients aged 60 and over in an outpatient onco-hematology clinic at a university hospital in Brazil)	Depression (10.2%)	*Benzodiazepine (10.5%) *Tricyclic antidepressant (TCA) and Serotonin reuptake inhibitors (SSRI) (7.6%)	*Diazepam, *Amitriptyline, Nortriptyline. and Paroxetine	The prevalence of potential inappropriate use of medicines (PIM) among elderly outpatients in oncology is 48.1% The most commonly used PIMs include benzodiazepines (10.5%) and antidepressants (7.6%)	NI
Reinert et al., 2015 Brazil	*n* = 56 (Cancer patients seen at the oncology clinic)	Depression (26.7%)	*Serotonin reuptake inhibitors (SSRI) *Tricyclic antidepressants (TCA) *Serotonin and Norepinephrine Reuptake Inhibitors (SNRI)	*Fluoxetine, Escitalopram, Paroxetine, *Amitriptyline, Imipramine. *Venlafaxine	Half of patients who screened positive for depression received antidepressant treatment 53.3% (8 of 15 depressed patients)	Beck Depression Inventory-II (BDI-II), for assessment of depressive symptoms cutoff point of 20 points was chosen
Zhao et al., 2014 China	*n* = 460 (Patients hospitalized for cancer were recruited from the oncology department of a university hospital in Beijing, China)	Depressive disorders (25.9%): *Major depressive disorder (MDD): 12.6 *Dysthemia: 9.1 *Minor depressive disorder: 1.7 *Mood disorder: 2.4	Antidepressants (25.9%)	NI	Only 3.4% of depressed patients were recognized by their oncologists, just 2.5% received treatment, and 0.8% were referred for psychiatric care	The Mini International Neuropsychiatric Interview 5.0 by eight trained psychiatrists
Ng et al., 2014 Malaysia	*n* = 3345 (Oncology patients between 2008 and 2012)	NI	Anxiolytics and hypnotics (12.3%), Antidepressants (7.8%) Antipsychotics (5.6%)	NI	Use of Psychotropic Drugs (12.6%)	NI

*NI* not indicated

Not all percentages of psychoactive medication classes used and percentages of patients with psychiatric diagnosis or psychiatric symptoms were reported in the selected studies

Asterisks (*) were used to list medications (column 5) and their respective psychoactive classes (column 4)

### Factors influencing the prescription and use of psychoactive medications among cancer

Several factors influenced the prescription and the use of psychoactive medications among cancer patients. Comorbidities were common in this population and were associated with the use of potentially inappropriate medications (PIMs). According to Tian et al., older cancer patients with comorbidities faced a higher risk of using PIMs, with a prevalence of 32.7%, and benzodiazepines were the most frequently prescribed PIMs [19]. Furthermore, cancer patients with comorbidities who took five or more medications daily (polypharmacy) were more likely to use potentially inappropriate psychoactive medications, as highlighted by Reis et al. [23].

Socioeconomic factors also contributed to disparities in psychoactive drug use among cancer patients. Geographic and health insurance variations play an important role. Bai et al. reported that cancer patients covered by Urban Employee Basic Medical Insurance (UEBMI) are more likely to use psychotropic medications compared to those insured under Urban Rural Resident Basic Medical Insurance (URRBMI) (OR = 1.18, 95% CI = 1.15–1.20). Additionally, there was a notable regional imbalance within the same country, with a significantly higher prevalence of psychoactive drug use observed among cancer patients in the Eastern region of China compared to the Western region (OR = 2.33, 95% CI = 2.27–2.40) [22].

These disparities are further compounded by the under-recognition and under-treatment of depression in cancer patients, as highlighted by several studies. For instance, Zhao et al. observed a high prevalence and a very low recognition rate of depression among Chinese cancer inpatients [25]. In Brazil, a study revealed that only half of the patients who screened positive for depression using the Beck Depression Inventory-II (BDI-II) received appropriate antidepressant treatment [24].

Ng et al. suggested that low prescription rates of psychoactive medications could be due to the underdiagnosis of depression or the increased caution in prescribing such drugs to cancer patients [26]. Lam et al. reinforced this notion, reporting that the low proportion of documented psychiatric diagnoses may reflect significant underdiagnosis of mental disorders in this population [18]. In India, only a quarter of psychoactive prescriptions were initiated by psychiatrists, with most prescriptions being driven by the need to manage specific symptoms rather than addressing underlying psychiatric conditions [13].

## Discussion

The prescription and use of psychoactive medications among cancer patients highlight the heavy burden of mental health conditions such as anxiety, depression, sleep disturbances, and other psychotic disorders within this population [28]. These conditions severely impact the cancer patient’s quality of life, leading to a notable decline in overall well-being and negatively affecting treatment outcomes [29]. Our findings reveal a considerable variability in the prescription and use of these medications, influenced by a range of clinical, systemic, and sociocultural factors.

Across studies, the use of psychoactive medication varied widely, from frequent use among patients with comorbidities in some cases to underuse in others [18, 26]. Prescriptions were sometimes symptom-driven rather than diagnosis-based [13]. Additional concerns emerged regarding potentially inappropriate prescribing in older adults [20]. Moreover, evidence from the Brazilian study revealed that only about half of patients with depression received adequate treatment [24].

In high-income countries, cancer survivors show a higher use of psychotropic medications compared to the general population. In the United States, 17–19% of cancer adults under 65 and 15–16% of those aged 65 and older report using such drugs, versus only 9–12% among adults without a cancer history. Antidepressants represent the most frequently prescribed class, followed by anti-anxiety medications [30]. Comparable trends have been observed in the Netherlands, where psychotropic prescriptions are frequent among cancer patients and tend to rise further in the terminal stage of illness [31].

In LMICs, despite the high prevalence of depression and anxiety, these disorders remain frequently underdiagnosed among cancer patients, particularly in those with advanced disease ​[32]. This underdiagnosis is particularly concerning, as psychological distress in cancer patients can exacerbate physical symptoms and affect overall quality of life. Several barriers contribute to this under recognition, including the lack of integration between oncology and mental health services, and limited access to mental health professionals, issues that are especially pronounced in low- and middle-income countries [11].

Additionally, cultural beliefs, such as the perception that cancer is a form of divine punishment, and stigma surrounding mental health can contribute to the underdiagnosis of mental health disorders [25, 33, 34]. In many African settings, mental illness is also interpreted as spiritual possession or personal weakness, making patients less likely to report symptoms or seek formal care. Instead, many turn to traditional healers or religious leaders before presenting to medical services [35, 36].

Furthermore, older cancer patients with comorbid conditions are particularly vulnerable to potentially inappropriate medication (PIM) use. Managing multiple medications increases the risk of drug interactions, adverse effects, and inappropriate prescribing. Psychoactive drugs such as benzodiazepines and antidepressants, though commonly prescribed for anxiety or depression, can pose significant risks in the elderly, including cognitive decline and increased risk of falls [19, 20, 23].

Socioeconomic and structural factors also contribute to disparities in psychoactive medication use. Geographic location is a key determinant, with patients in urban areas more likely to have access to comprehensive care, specialized providers, and mental health services. In contrast, rural populations often face delayed diagnosis, limited treatment options, and restricted access to medications and psychosocial interventions [37, 38].

Similarly, disparities in health insurance coverage influence access to treatment. Patients with more comprehensive insurance plans, such as those typically offered to urban employees, are more likely to benefit from a broader range of healthcare services, including access to medications for mental health conditions. Conversely, those covered by more basic insurance plans, such as the URRBMI, which is often the case for rural populations or low-income groups, may face higher out-of-pocket costs [22, 39, 40]. This is further aggravated by the limited availability of psychotropic medications in public health systems of LMICs, where essential psychiatric drugs are frequently out of stock or only available in urban hospitals. Affordability is also a barrier, especially when prescriptions require out-of-pocket payment in the absence of universal mental health coverage [41].

Healthcare system inequalities further aggravate the situation. In regions where mental health is not integrated into cancer care, or where there is a shortage of trained professionals, patients may not receive psychological assessments or treatment, including the prescription of appropriate psychoactive medications [42, 43].

Both patients and healthcare providers may face perceptual barriers related to the use of psychoactive medications. Concerns about potential side effects, risks of dependency, and insufficient understanding of their therapeutic role can affect the acceptance, prescription, and adherence to these treatments in cancer care [44].

Moreover, there is a lack of reliable mental health data in many LMICs, limiting the ability to develop effective public health policies and allocate resources appropriately. Mental health is often deprioritized in national healthcare agendas, and systems for surveillance or psychiatric registries are rarely established [11, 45]. In contrast to high-income countries, where large-scale and high-quality epidemiological studies provide valuable insights into prescription patterns, similar data are largely lacking in LMICs. This gap limits the ability to comprehensively assess psychoactive medication use and prescribing practices in LMICs. It is also important to note that some regions of the world are not represented, notably the Middle East, Europe, and Africa, which include several low- and middle-income countries (LMICs). Although the countries included in this review share to some extent similar structural challenges in psycho-oncology care (access to diagnostic facilities, access to and availability of medicines) [46–48], these findings are country-specific and should not be interpreted as capturing the full diversity of middle-income countries.

This study has several strengths and limitations. One strength is its comprehensive analysis of factors influencing psychoactive medications’ use and prescription in cancer patients in resource-limited countries, including clinical, social, and economic aspects, as well as geographic and insurance disparities. However, a limitation is that we were unable to find studies from low-income countries, where access to healthcare is more restricted. Additionally, as a systematic review, we relied on existing literature, which may not fully capture individual patient experiences, such as personal beliefs and attitudes toward mental health that could also impact medication use. Furthermore, the review lacked access to some data, such as patient-reported outcomes, which could have provided more insights about patient perspectives.

## Conclusion

In conclusion, this review highlights the variability and complexity of psychoactive medications prescribing and use among cancer patients. Their use is shaped by multiple factors, including comorbidities, polypharmacy in older adults, and socioeconomic disparities that limit access to healthcare and medicines.

Future research could focus on longitudinal or qualitative studies exploring the personal, cultural, and contextual factors influencing mental health treatment in cancer patients, offering deeper insights into their experiences and needs. In addition, strengthening mental health awareness and prevention, alongside regular medication reviews especially for older adults could help reduce the use of potentially inappropriate medications and minimize harmful drug interactions. Addressing these gaps has the potential to improve both psychological well-being and clinical outcomes, contributing to more comprehensive and equitable cancer care.

## Supplementary Information

Below is the link to the electronic supplementary material.

Supplementary Material 1 (PDF 47.4 KB)

Supplementary Material 2 (PDF 117 KB)

## Data Availability

No datasets were generated or analysed during the current study.

## References

[1] Pramesh CS, Badwe RA, Bhoo-Pathy N, Booth CM, Chinnaswamy G, Dare AJ et al (2022) Priorities for cancer research in low- and middle-income countries: a global perspective. Nat Med 28(4):649–657 35440716 10.1038/s41591-022-01738-x PMC9108683

[2] Cancer |WHO | (2024) Regional Office for Africa. Available from: https://www.afro.who.int/health-topics/cancer. Accessed 4 Oct 2024

[3] Ostovar S, ModarresiChahardehi A, MohdHashim IH, Othman A, Kruk J, Griffiths MD (2022) Prevalence of psychological distress among cancer patients in Southeast Asian countries: a systematic review. Eur J Cancer Care (Engl) 31(6):e13669 35934684 10.1111/ecc.13669 PMC9786346

[4] Ebob-Anya BA, Bassah N (2022) Psychosocial distress and the quality of life of cancer patients in two health facilities in Cameroon. BMC Palliat Care 21(1):96 35650571 10.1186/s12904-022-00981-w PMC9158288

[5] Benton JZ, Bergerot CD, Woodruff P, Williams SB, Wallis CJD, Klaassen Z (2022) Mental health screening and diagnosis in cancer patients: impact on mortality and suggestion of racial bias. Cancer 128(2):234–236 34550606 10.1002/cncr.33901

[6] Walker ZJ, Xue S, Jones MP, Ravindran AV (2021) Depression, anxiety, and other mental disorders in patients with cancer in low- and lower-middle–income countries: a systematic review and meta-analysis. JCO Glob Oncol 7:1233–1250 34343029 10.1200/GO.21.00056 PMC8457869

[7] Nakie G, Melkam M, Takelle GM, Fentahun S, Rtbey G, Andualem F et al (2024) Depression, anxiety and associated factors among cancer patients in Africa; a systematic review and meta-analysis study. BMC Psychiatry 24(1):939 39716105 10.1186/s12888-024-06389-5 PMC11668085

[8] Ungvari Z, Fekete M, Buda A, Lehoczki A, Fekete JT, Varga P et al (2025) Depression increases cancer mortality by 23–83%: a meta-analysis of 65 studies across five major cancer types. GeroScience. Available from: 10.1007/s11357-025-01676-9. Accessed 22 Sep 2025 10.1007/s11357-025-01676-9 PMC12972437 40314846

[9] Depression symptoms (2024) How to manage depression. Available from: https://www.cancer.org/cancer/managing-cancer/side-effects/emotional-mood-changes/depression.html. Accessed 24 Oct 2024

[10] Park SJ, Wai A, Pavithran K, Kunheri B, Valsraj K (2021) Cancer and severe mental illness in low- and middle-income countries: the challenges and outlook for the future. Psychooncology 30(12):2002–2011 34449954 10.1002/pon.5796

[11] Rathod S, Pinninti N, Irfan M, Gorczynski P, Rathod P, Gega L et al (2017) Mental health service provision in low- and middle-income countries. Health Serv Insights 28(10):1178632917694350 10.1177/1178632917694350 PMC5398308 28469456

[12] Venkataramu VN, Ghotra HK, Chaturvedi SK (2022) Management of psychiatric disorders in patients with cancer. Indian J Psychiatry 64(Suppl 2):S458 35602367 10.4103/indianjpsychiatry.indianjpsychiatry_15_22 PMC9122176

[13] Mohamed F, Uvais NA, Moideen S, Cp RR, Saif M (2024) Psychotropic medication prescriptions for home-based palliative care oncology patients. Prim Care Companion CNS Disord 26(2):23m03668 38728674 10.4088/PCC.23m03668

[14] Riechelmann R, Girardi D (2016) Drug interactions in cancer patients: a hidden risk? J Res Pharm Pract 5(2):77–78 27162799 10.4103/2279-042X.179560 PMC4843587

[15] Moher D, Liberati A, Tetzlaff J, Altman DG (2009) PRISMA Group. Preferred Reporting Items for Systematic Reviews and Meta-analyses: the PRISMA statement. BMJ 339 2535 PMC3090117 21603045

[16] Wells GA, Shea B, O’Connell D, Peterson J, Welch V, Losos M, Tugwell P (2000). Ottawa Hospital Research Institute. Available from: https://www.ohri.ca/programs/clinical_epidemiology/oxford.asp. Accessed 13 Nov 2024

[17] Modesti PA, Reboldi G, Cappuccio FP, Agyemang C, Remuzzi G, Rapi S et al (2016) Panethnic differences in blood pressure in Europe: a systematic review and meta-analysis. PLoS ONE 11(1):e0147601 26808317 10.1371/journal.pone.0147601 PMC4725677

[18] Lam CS, Lee CP, Chan JWY, Cheung YT (2024) Patterns and factors associated with the prescription of psychotropic medications after diagnosis of cancer in Chinese patients: a population-based cohort study. Pharmacoepidemiol Drug Saf 33(2):e5754 38362653 10.1002/pds.5754

[19] Tian F, Yang R, Chen Z, Duan X, Yuan P (2022) The prevalence and factors associated with potentially inappropriate medication use in Chinese older outpatients with cancer with multimorbidity. J Geriatr Oncol 13(5):629–634 35183489 10.1016/j.jgo.2022.02.006

[20] Machado TRL, Menezes de Pádua CA, Drummond PL de M, Silveira LP, Malta JS, Santos RMMD et al (2022) Use of fall risk-increasing drugs in older adults with multiple myeloma: a cross-sectional study. J Geriatr Oncol 13(4):493–8 10.1016/j.jgo.2022.01.007 35086797

[21] Pu B, Wang N, Wang C, Sun B (2022) Clinical observation on the benefits of antidepressant intervention in advanced cancer patients. Medicine (Baltimore) 101(26):e29771 35776994 10.1097/MD.0000000000029771 PMC9239637

[22] Bai L, Xu Z, Huang C, Sui Y, Guan X, Shi L (2020) Psychotropic medication utilisation in adult cancer patients in China: a cross-sectional study based on national health insurance database. Lancet Reg Health - West Pac 1(5):100060 10.1016/j.lanwpc.2020.100060 PMC8315446 34327398

[23] Reis CM, Dos Santos AG, de Jesus Souza P, Reis AMM (2017) Factors associated with the use of potentially inappropriate medications by older adults with cancer. J Geriatr Oncol 8(4):303–307 28602709 10.1016/j.jgo.2017.05.003

[24] Reinert CdeA, Ribas MR, Zimmermann PR (2015) Drug interactions between antineoplastic and antidepressant agents: analysis of patients seen at an oncology clinic at a general hospital. Trends Psychiatry Psychother 37(2):87–93 26222300 10.1590/2237-6089-2015-0003

[25] Zhao L, Li X, Zhang Z, Song C, Guo C, Zhang Y et al (2014) Prevalence, correlates and recognition of depression in Chinese inpatients with cancer. Gen Hosp Psychiatry 36(5):477–482 24961793 10.1016/j.genhosppsych.2014.05.005

[26] Ng CG, Mohamed S, Wern TY, Haris A, Zainal NZ, Sulaiman AH (2014) Comparison of psychotropic prescriptions between oncology and cardiology inpatients: result from a pharmacy database in a teaching hospital in Malaysia. Asian Pac J Cancer Prev APJCP 15(10):4261–4264 24935381 10.7314/apjcp.2014.15.10.4261

[27] Tamura BK, Bell CL, Inaba M, Masaki KH (2012) Outcomes of polypharmacy in nursing home residents. Clin Geriatr Med 28(2):217–236 22500540 10.1016/j.cger.2012.01.005

[28] Więckiewicz G, Weber S, Florczyk I, Gorczyca P (2024) Socioeconomic burden of psychiatric cancer patients: a narrative review. Cancers 16(6), 1108. https://www.mdpi.com/2072-6694/16/6/1108. Accessed 11 Dec 2024 10.3390/cancers16061108 PMC10969503 38539443

[29] Pinquart M, Duberstein PR (2010) Depression and cancer mortality: a meta-analysis. Psychol Med 40(11):1797 20085667 10.1017/S0033291709992285 PMC2935927

[30] Punekar RS, Short PF, Moran JR (2012) Use of psychotropic medications by U.S. cancer survivors. Psychooncology 21(11):1237–1243 21905155 10.1002/pon.2039 PMC4079257

[31] Ng CG, Boks MP, Smeets HM, Zainal NZ, de Wit NJ (2013) Prescription patterns for psychotropic drugs in cancer patients; a large population study in the Netherlands. Psychooncology 22(4):762–767 22351591 10.1002/pon.3056

[32] Pitman A, Suleman S, Hyde N, Hodgkiss A (2018) Depression and anxiety in patients with cancer. BMJ 25(361):k1415 10.1136/bmj.k1415 29695476

[33] Yeo SS, Meiser B, Barlow-Stewart K, Goldstein D, Tucker K, Eisenbruch M (2005) Understanding community beliefs of Chinese-Australians about cancer: initial insights using an ethnographic approach. Psychooncology 14(3):174–186 15386778 10.1002/pon.831

[34] Tang L, de Groot J, Bultz BD (2009) Psychosocial oncology in China. Chin-Ger J Clin Oncol 8(3):123–128

[35] Ae-Ngibise K, Cooper S, Adiibokah E, Akpalu B, Lund C, Doku V et al (2010) “Whether you like it or not people with mental problems are going to go to them”: a qualitative exploration into the widespread use of traditional and faith healers in the provision of mental health care in Ghana. Int Rev Psychiatry Abingdon Engl 22(6):558–567 10.3109/09540261.2010.536149 21226644

[36] Mendenhall E, De Silva MJ, Hanlon C, Petersen I, Shidhaye R, Jordans M et al (2014) Acceptability and feasibility of using non-specialist health workers to deliver mental health care: stakeholder perceptions from the PRIME district sites in Ethiopia, India, Nepal, South Africa, and Uganda. Soc Sci Med 1982(118):33–42 10.1016/j.socscimed.2014.07.057 PMC4167946 25089962

[37] Shawahna R, Debay M, Rahman N ur (2014) Inequalities in health care and behaviour in patients with diabetes and concurrent hypertension in Lahore, Pakistan. Tanzan J Health Res 15(4). Available from: http://www.ajol.info/index.php/thrb/article/view/89747. Accessed 17 Apr 2024 10.4314/thrb.v15i4.5 26591699

[38] Iamtrakul P, Chayphong S, Gao W (2024) Assessing spatial disparities and urban facility accessibility in promoting health and well-being. Transp Res Interdiscip Perspect 1(25):101126

[39] Meng Q, Fang H, Liu X, Yuan B, Xu J (2015) Consolidating the social health insurance schemes in China: towards an equitable and efficient health system. Lancet Lond Engl 386(10002):1484–1492 10.1016/S0140-6736(15)00342-6 26466052

[40] Ma X, Oshio T (2020) The impact of social insurance on health among middle-aged and older adults in rural China: a longitudinal study using a three-wave nationwide survey. BMC Public Health 1(20):1842 10.1186/s12889-020-09945-2 PMC7709307 33261609

[41] World Health Organization, Fundação Calouste Gulbenkian (2017) Improving access to and appropriate use of medicines for mental disorders. Geneva: World Health Organization 95 p. Available from: https://iris.who.int/handle/10665/254794. Accessed 11 Jul 2025

[42] Lawrence D, Kisely S (2010) Inequalities in healthcare provision for people with severe mental illness. J Psychopharmacol Oxf Engl. 24(4_suppl), 61–8 10.1177/1359786810382058 PMC2951586 20923921

[43] Deshields T, Asvat Y (2023) The case for accelerating integrated mental health care in the cancer setting. JCO Oncol Pract 19(5):231–233 36800568 10.1200/OP.22.00840

[44] Mosher CE, Winger JG, Hanna N, Jalal SI, Fakiris AJ, Einhorn LH et al (2014) Barriers to mental health service use and preferences for addressing emotional concerns among lung cancer patients. Psychooncology 23(7):812–819 24493634 10.1002/pon.3488 PMC4082441

[45] Acuña-Rodríguez MP, Fiorillo-Moreno O, Montoya-Quintero KF, Tejan Mansaray F (2025) Mental Health Workforce Inequities Across Income Levels: Aligning Global Health Indicators, Policy Readiness, and Disease Burden. Psychol Res Behav Manag 1449–1454. https://www.who.int/publications/i/item/9789240036703. Accessed 11 Jul 2025 10.2147/PRBM.S532912 PMC12182244 40548171

[46] Shi Y, Zhang L, Fu J, Shao R, Yan J (2023) An analysis of accessibility of representative psychotropic medicine from the World Health Organization model list of essential medicines in developing countries with different income levels. Value Health 26(4):528–535 36442833 10.1016/j.jval.2022.11.009

[47] Barbui C (2015) Access and use of psychotropic medicines in low-resource settings. Epidemiol Psychiatr Sci 24(3):206–209 25787227 10.1017/S2045796015000268 PMC6998629

[48] Saraceno B, van Ommeren M, Batniji R, Cohen A, Gureje O, Mahoney J et al (2007) Barriers to improvement of mental health services in low-income and middle-income countries. The Lancet 370(9593):1164–1174 10.1016/S0140-6736(07)61263-X 17804061

