# Supplementary material for: Prescription and use of psychoactive medications among cancer patients and associated factors in lower and upper middle-income countries: systematic review
Source: Support Care Cancer. 2026 Jan 6;34(1):67. doi: 10.1007/s00520-025-10272-8 (PMC12769504; doi:10.1007/s00520-025-10272-8)
Supplement: Supplementary file 1 — Supplementary Material 1 (PDF 47.4 KB) [file 520_2025_10272_MOESM1_ESM.pdf]

Online Resource 1

Quality assessment of selected cohort studies using **the Newcastle-Ottawa Scale (NOS):** Domain-specific and overall scores.

	Newcastle-Ottawa Scale (NOS) scores				
**Authors, years**	**Selection**	**Comparability**	**Outcome**	**Overall (out of 9)**	***Quality score**
Ng et al, 2014	4	2	2	8	Good
Lam et al, 2024	4	1	2	7	Good

*Quality score: studies were assigned an overall quality rating of "good", "fair", or "poor".

For the purpose of this assessment, each star (★) is considered equivalent to one point.

A study was considered good quality if it received 3 to 4 stars (★) in the selection domain, 1 to 2 stars in the comparability domain, and 2 to 3 stars in the outcome/exposure domain.

Fair quality was assigned to studies with 2 stars in the selection domain, 1 to 2 stars in the comparability domain, and 2 to 3 stars in the outcome/exposure domain.

A study was rated as poor quality if it received either 0 to 1 star in the selection domain, 0 stars in the comparability domain, or 0 to 1 star in the outcome/exposure domain.

Quality assessment of selected cross-sectional studies using **the adapted version of Newcastle-Ottawa Scale (NOS) for cross-sectional studies**: Domain-specific and overall scores.

	Newcastle-Ottawa Scale (NOS) scores				
**Authors, years**	**Selection**	**Comparability**	**Outcome**	**Overall (out of 10)**	****Quality score**
Reis et al, 2017	3	2	2	7	Good
Zhao et al, 2014	3	2	3	8	Good
Bai et al, 2020	3	2	3	8	Good
Reinert et al, 2015	2	2	2	6	Fair
Tian et al, 2022	3	2	3	8	Good
Machado et al, 2022	3	2	2	7	Good
Mohamed et al, 2024	3	1	3	7	Good
Pu et al, 2022	2	2	2	6	Fair

**Quality Score: Studies were classified as "good," "fair," or "poor" based on their scores across different domains. For the purpose of this assessment, each star (★) is considered equivalent to one point.

Good quality: 3 to 4 stars (★) in the selection domain, 1 to 2 stars in the comparability domain, and 2 to 3 stars in the outcome/exposure domain.

Fair quality: 2 stars in the selection domain, 1 to 2 stars in the comparability domain, and 2 to 3 stars in the outcome/exposure domain.

Poor quality: 0 to 1 star in the selection domain, 0 stars in the comparability domain, or 0 to 1 star in the outcome/exposure domain.
