# Supplementary material for: Prescription and use of psychoactive medications among cancer patients and associated factors in lower and upper middle-income countries: systematic review
Source: Support Care Cancer. 2026 Jan 6;34(1):67. doi: 10.1007/s00520-025-10272-8 (PMC12769504; doi:10.1007/s00520-025-10272-8)
Supplement: Supplementary file 2 — Supplementary Material 2 (PDF 117 KB) [file 520_2025_10272_MOESM2_ESM.pdf]

Online resource 2: Overview and characteristics of included studies.

Authors	Year	Country	Study design	Study objective	Population	Socio-demographic characteristics	Study settings	Medical data sources	Type of cancer (rate %)
**Lam et al**	2024	China	Retrospective cohort	To examine the patterns and factors associated with the prescription of psychotropic drugs after a cancer diagnosis.	**n=1868**<br>cancer patients who have been prescribed psychotropic medications	**Age:** (mean ± SD)<br>58.7 ±14.5<br>**Gender distribution:**<br>Women: 54.9%<br>**Income level:**<br>low income: 40.2%	Population-based	Medical records were obtained from the Hospital Authority (HA) in Hong Kong.	Lip, oral cavity, pharynx.<br>Digestive organs.<br>Respiratory organs.<br>Bone, skin, soft tissues<br>Breast.<br>Female genital organs.<br>Male genital organs.<br>Urinary organs.<br>Eye, brain and other cancers.<br>Ill-defined, secondary and unspecified sites.<br>Lymphatic and hematopoietic tissue.
**Mohamed et al**	2024	India	Cross-sectional	To examine the complexities of psychotropic medication prescription in home-based palliative care for oncology patients.	**N=125**<br>Cancer patients registered for palliative care at home	**Age:** (mean ± SD)<br>64.4 ± 14.9<br>Women: 50.4%	Hospital based study	Medical records of patients receiving palliative home care for cancer in a tertiary hospital, Kozhikode, Kerala, India.	Breast cancer (14,4)<br>Lung cancer (13,6)<br>Ovarian cancer (5,6)<br>Prostate cancer (4,8)<br>Gall bladder cancer (4.8)<br>Colon cancer (4.8)<br>Others (52)
**Tian et al**	2022	China	Cross-sectional	To examine the prevalence and the predictors of potentially inappropriate medication use in Chinese older outpatients with cancer with multimorbidity in Chengdu.	**N=6160**<br>Outpatients aged 65 and over with cancer.	**Age:** Median (IQR)<br>72 (68-78)<br>**Gender distribution:**<br>Men: 53.47%	Hospital-based	Electronic medical data from nine tertiary hospitals in Chengdu from January 2018 December 2018.	Lung cancer (20.70)<br>Breast cancer (18.83)<br>Colorectal cancer (16.36)<br>Prostate cancer (12.76)<br>Gastric cancer (6.38)<br>Liver cancer (6.15)<br>Esophageal cancer (4.84)<br>Uterine cancer (2.11)<br>Kidney cancer (2.03)<br>Thyroid cancer (1.90)

**Machado et al**	2022	Brazil	Cross-sectional	To investigate the use of fall risk-increasing drugs (FRIDs) and associated factors and to assess the use of medicines that induce neuropathy in older adults with multiple myeloma (MM).	**n=153**<br>patients diagnosed with multiple myeloma and treated in the oncology and hematology departments of a capital city in south-east Brazil.	**Age:** Median (IQR) 70.9 (60-92)<br>**Gender distribution:** Women: 54.2%<br>**Income level:** 53.6% > three minimum wages (1 minimum wage =275 USD)<br>**Schooling:** 52.3% higher (high school or high education.<br>**Health service type:** private	Hospital-based	Socio-demographic data and the use of prescription and non-prescription medications was collected by interviewing patients in waiting room of the service. Information on antineoplastics and supportive therapy medications used was collected from the medical records.	Multiple myeloma<br>Stage 1: (31.4)<br>Stage 2: (26)<br>Stage 3: (31.4)<br>11% missing data
**Pu et al**	2022	China	Cross-sectional	To examine the effect of antidepressants on patients with advanced cancer and analyze their characteristics in order to explore the reasonable use of these drugs.	**n= 152**<br>Patients with advanced cancer who received antidepressant intervention with Sertraline (June 2018 to June 2020).	**Age**: (mean ± SD) 59.5 ± 5.2<br>**Gender distribution:** Women: 64.47%	Hospital-based	Medical records of patients hospitalized and treated with Setraline	Lung cancer (21.05)<br>Esophageal cancer (21)<br>Colorectal cancer (19.07)<br>Stomach cancer (13.81)<br>Breast cancer (11.18)<br>Head and neck cancer (6.57)
**Bai et al**	2020	China	Cross-sectional	To analyze the prevalence and potential predictors of psychotropic drug use in adult cancer patients in China	**n=48111**<br>Patients diagnosed with cancer were included in the study, after excluding patients who were diagnosed with psychiatric disorders, or were prescribed psychotropic medications prior to cancer diagnosis	**Age:** 18.7% (45-59)<br>**Gender distribution:** Women: 51.49%<br>**Region:** 49.92 % Eastern china<br>**Health insurance:** 72.98% UEBMI (Urban Employee Basic Medical Insurance)	Population-based	The China Health Insurance Association database. between 2015 and 2017, which contained information on healthcare use among beneficiaries of basic medical insurance.	Lip, oral cavity and pharynx<br>Digestive organs<br>Respiratory and intrathoracic organs<br>Breast<br>Female genital organs<br>Urinary tract<br>Thyroid and other endocrine glands<br>Ill-defined, secondary and unspecified sites<br>Lymphoid, haematopoietic and related tissues<br>Other.

**Reis et al**	2017	Brazil	Cross-sectional	To identify factors associated with potential inappropriate drug use in elderly cancer patients	**n=160**<br>Patients aged 60 and over in an outpatient onco-hematology clinic at a university hospital in Brazil	**Age:** Median (IQR) 67.5 (10)<br>**Gender distribution:** Women: 57.5%	Hospital-based study	Socio-demographic variables and prescribed and non-prescribed medications were recorded during patients' interview. The antineoplastic medication and medication used for supportive therapy were recorded in the chemotherapy prescription.	Breast (28.1)<br>Colorectal (22.5)<br>Lung (7.5)<br>Stomach (6.9)<br>Prostate (6.3)<br>Oesophagus (4.4)<br>Hematologic neoplasia myelomas (1.2)<br>Lymphomas (0.6)<br>Leukemias (0.6)<br>Others (21.9)
**Reinert et al**	2015	Brazil	Cross-sectional	To identify simultaneous use of antineoplastic and antidepressant agents.	**n=56**<br>Cancer patients seen at the oncology clinic	**Age:** mean 56.5<br>**Gender distribution:** Women: 75%	Hospital-based study	Interviewing patients and reviewing medical records	Breast cancer (25)<br>Digestive system cancers (21.4)<br>Other cancers (10.7)<br>Oropharynx cancers (8.9)<br>Melanoma (8.9)<br>Gynecological cancers (7.1)<br>Lung cancer (5.3)<br>Prostate cancer (5.3)<br>Lymphoma (5.3)
**Ng et al**	2014	Malaysia	Retrospective cohort	To examine the prescription rates in cancer patients of three common psychotropic drugs: anxiolytic/hypnotic, antidepressant and antipsychotic	**n=3345**<br>Oncology patients between 2008 and 2012	**Age:** mean 57 years old<br>**Gender distribution:** Women: 69%	Hospital-based study	The University of Malaya Medical Centre (UMMC) pharmacy database.	Breast cancer (32.9%)<br>Liver cancer (28.6%)<br>Lung cancer (26%)<br>Uterine cancer (23.1%)<br>Ovarian cancer (20.6)<br>Digestive cancers (19.4%)

**Zhao et al**	2014	China	Cross-sectional	To determine the prevalence, correlations and recognition rates of depressive disorders in Chinese patients hospitalized for cancer and to identify treatment of people suffering from depressive disorders.	**N=460**<br>Patients hospitalized for cancer were recruited from the oncology department of a university hospital in Beijing, China.	**Age:** (mean ± SD)<br>59.4 ± 12<br>**Gender distribution:**<br>Women: 50.87%	Hospital-based study	Clinical variables and data on the recognition of depression by oncologists were obtained by reviewing patients' medical records, unclear or inaccurate information was further discussed with the patients and/or their treating physicians.	Cancer without precision of the type.
